# Lactate dehydrogenase and creatine kinase as poor prognostic factors in lung cancer: A retrospective observational study

**DOI:** 10.1371/journal.pone.0182168

**Published:** 2017-08-02

**Authors:** Lei Liu, Ying He, Ge Ge, Lei Li, Ping Zhou, Yihan Zhu, Huairong Tang, Yan Huang, Weimin Li, Li Zhang

**Affiliations:** 1 Laboratory of Pathology, West China Hospital, Sichuan University, Chengdu, Sichuan Province, China; 2 Key Laboratory of Transplantation Engineering and Immunology, Ministry of Health, West China Hospital, Sichuan University, Chengdu, Sichuan Province, China; 3 West China Medical School, Sichan University, Cheng Du, Sichuan Province, China; 4 Department of Respiratory, West China Hospital, Sichuan University, Cheng Du, Sichuan Province, China; 5 Health Management Center, West China Hospital, Sichuan University, Cheng Du, China; Roswell Park Cancer Institute, UNITED STATES

## Abstract

**Purpose:**

Circulating molecules play important roles in lung cancer diagnosis. In addition, plasma lactate dehydrogenase (LDH) and creatine kinase (CK) have been shown to be closely related to tumor progression in breast cancer, prostate cancer, and colonel cancer. However, the relationships between LDH and CK levels with metastasis occurrence and the survival status of lung cancer patients remain unclear.

**Experimental design:**

A total of 1142 lung cancer patients were enrolled in this study and were separated into negative or positive groups, according to the plasma levels of CK or LDH. Patients in both groups were assessed for clinical characteristics, metastasis occurrence, and survival status. The Cox regression model was then introduced to confirm whether CK and LDH could act as independent factors for predicting a poor prognosis.

**Results:**

The results indicated that CK had a close relationship with bone (p < 0.05) and lymph node (p < 0.05) metastases. In addition, LDH was strongly related with bone (p < 0.05), adrenal gland (p < 0.05), and lymph node (p < 0.05) metastases. CK and LDH were also correlated with the survival status of the lung cancer patients (all p < 0.001). According to specific histological classification analysis, it was found that CK was closely related to the survival status of adenocarcinoma (ADC) and squamous cell carcinoma (SCC) patients, while LDH was only correlated with that of ADC patients. Cox regression analysis confirmed that CK and LDH could act as independent factors for predicting a poor prognosis in ADC but not SCC patients.

**Conclusions:**

For the first time, our study confirmed the role of CK in metastasis occurrence and the survival status of lung cancer patients. In addition, it also demonstrated that CK and LDH could be used as independent factors to predict a poor prognosis in ADC patients. The identification of CK and LDH will play important roles in lung cancer diagnosis and poor outcome prediction in the future.

## Introduction

Lung cancer is one of the most common cancer-related deaths worldwide, accounting for 86,380 male and 71,660 female deaths in the United States in 2015 [1]. The mortality due to lung cancer in China is 610.2 per 100,000 [2]. Moreover, the average 5-year survival rate for lung cancer is less than 15% due to late diagnosis [3]. Furthermore, the increased aging population and environmental pollution have enhanced the morbidity of lung cancer [4].

The current noninvasive diagnostic methods for lung cancer used in clinical practice include X-ray and computed tomography (CT) scans [5]. The National Lung Screening Trial (NLST), conducted in the United States, enrolled 53,454 participants. All the participants were randomly divided into a low-dose CT scan group (26,722 cases) or a chest radiography group (26,732 cases), and they all underwent annual screening. The results indicated that although the low-dose CT scan group had a 20% reduced mortality compared with the X-ray group, 96.4% of those in the low-dose CT scan group and 94.5% of those in the radiography group who had a positive diagnosis of lung cancer were found to be false positives [6]. In addition to CT and X-ray, other diagnostic methods for lung cancer are invasive, painful, and time-consuming, such as bronchoscopy and needle biopsy. Therefore, the discovery of noninvasive, nonradioactive, and rapid diagnostic methods for lung cancer is urgently needed [7].

At present, numerous tumor-related molecules, produced by cancer cells or the tumor microenvironment, have been identified in the plasma of lung cancer patients; these molecules can be used for diagnosing and monitoring disease recurrence [8]. Circulating proteins as lung cancer biomarkers, including carcinoembryonic antigen [9], Cyfra21-1 [10], CA199 [11], and CA125 [12], have been widely used in lung cancer diagnosis. However, these biomarkers have low sensitivities of 50–60% [12]. Increasing evidence has suggested that circulating lactate dehydrogenase (LDH) can predict the survival status in various cancers, including lung cancer [13], and overall survival in advanced non-small cell lung cancer (NSCLC) [14]. Creatine kinase (CK) also has been associated closely with breast cancer development and progression [15]. Moreover, inactivation of CK has been strictly correlated with oral squamous cell carcinoma (SCC) progression [16]. However, systematically analyzing the relationships between LDH and CK levels with metastasis occurrence and the survival status in lung cancer patients have not yet been reported.

In this retrospective observational study, we measured the plasma levels of CK and LDH in 1142 patients to explore whether plasma CK and LDH levels could be used as independent factors for predicting a poor prognosis in lung cancer patients.

## Methods

### Ethics statement

The current research protocol was approved by the Medical Ethics Committee of West China Hospital. Written consent for sampling, study, and publication was obtained from each of the included patients.

Due to the retrospective nature of the study, the informed consent of participants in these study could be waived. Parts of the survival data were obtained through phone calls, so it’s difficult to get written consent. only verbal informed consent were obtained from these subjects or their legal guardians, only for those survival data were followed up via outpatient visit, written informed consents were obtained.

Institutional review board approval for this study was also obtained from West China Hospital. All the methods used in this study were followed in accordance with the approved protocols.

### Patients included in this study

This was a single-center, retrospective observational study that was conducted at the West China Hospital of Sichuan University, a tertiary academic hospital in western China. A total of 1142 lung cancer patients pathologically diagnosed in West China Hospital, from January 2008 to July 2012 with complete medical records and follow-up information, were enrolled in this study. Patients were excluded based on the exclusion criteria of without LDH and CK assays; without pathological confirmation of lung cancer; without evidence of metastasis; and lack of follow-up data.

### Procedures

All clinical information was extracted from the medical records within 2 weeks of the date of diagnosis. The occurrence of metastasis was confirmed according to the reports of whole-body and bone CT scans and/or lymph node biopsy. The date of death was obtained during a follow-up visit or telephone inquiry. The overall survival time was calculated from the date of diagnosis to death or the last follow-up visit. Plasma concentrations of LDH and CK were measured by immunoassays in the Department of Medical Laboratory, prior to surgery or any other treatment. The cut-off values for positive LDH and CK were 215 U/L and 60 U/L, respectively.

### Study design

Based on the cut-off values of LDH and CK, all enrolled lung cancer patients were divided into negative and positive groups. First, correlation analysis was performed to analyze the association between the LDH or CK level with clinical characteristics and metastatic status, respectively, in all lung cancer patients. The survival function was then analyzed to reveal the relationship between LDH or CK and survival status. Finally, the Cox regression model was used to confirm whether LDH and CK could act as independent prognostic factors for poor outcomes in lung cancer patients.

### Statistical methods for data analysis

SPSS 19.0 software was used in this study. Significant differences were determined using the chi-squared test. The Kaplan—Meier test was introduced to calculate the survival conditions in the negative and positive groups. The log-rank test was used to compare the differences of survival status between the two groups. The Cox regression model for multivariate analysis was conducted to determine the association between the LDH or CK level with clinical characteristics, metastasis occurrence, and survival status; to evaluate the hazards ratio (HR) for the negative and positive groups of LDH and CK; and to identify the independent predictors of poor outcomes in lung cancer patients.

http://dx.doi.org/10.17504/protocols.io.ir7cd9n

## Results

### CK and LDH levels were significantly correlated with metastases in lung cancer patients

A total of 1142 lung cancer patients were enrolled in this study and divided into negative and positive groups, according to the cut-off values of plasma CK and LDH. The demographic and clinical features of all enrolled patients are listed in Table 1. For the CK stratification analysis, including 678 cases in the positive group and 464 in the negative group, the results indicated that CK was closely related to the gender (p < 0.05) and tumor stage (p < 0.001). The reverse relationship was observed in bone (p < 0.05) and lymph node (p < 0.05) metastases by multiple metastasis analysis, which indicated that an increased plasma level of CK meant decreased numbers of metastasis occurrences (bone: 22.6% of CK-negative patients vs. 14.9% of CK-positive patients; lymph node: 60.6% of CK-negative patients vs. 54.3% of CK-positive patients) (Table 2).

**Table 1:** Demographics and clinical characteristic of enrolled lung cancer patients.

Characteristics	No. of patients (%) Total(1142)
**Gender**	
Male Female	790 (69.2) 352 (30.8)
**Age**	
<45 45–60 >60	97(8.5) 463 (40.5) 582 (51.0)
**Histological classification**	
Adenocarcinoma Squamous SCLC Others	307 (26.9) 608 (53.2) 170 (14.9) 57 (5.0)
**Stages**	
I II III IV	107 (9.4) 108 (9.5) 304 (26.6) 623 (54.5)
**Smoke status**	
No Yes	492 (43.1) 650 (56.9)
**Metastasis**	
No Yes	312 (27.3) 830 (72.7)
**CK**	
Negative Positive	678 (59.4) 464 (40.6)
**LDH**	
Negative Positive	732 (64.1) 410 (35.9)

Abbreviations: SCLC, Small Cell Lung Cancer; CK, Creatine Kinase; LDH, lactate dehydrogenase.

**Table 2:** Association of clinical characteristics and metastasis after stratification analysis by plasma CK levels.

	Negative n = 678	Positive n = 464	Total n = 1142	*p* value
**Basic Characteristic**				
**Age**				
<45 years 45–60 years >60 years	62(9.1%) 274(40.4%) 342(50.5%)	35(7.5%) 189(40.8%) 240(51.7%)	97 463 582	0.629
**Gender**				
Male Female	443(65.3%) 235(34.7%)	347(74.8%) 117(25.2%)	790 352	*<0*.05*
**Histological classification**				
Adenocarcinoma Squamous SCLC Others	190(28.0%) 366(54.0%) 91(13.4%) 31(4.6%)	117(25.2%) 242(54.2%) 79(17.0%) 26(5.6%)	307 608 170 57	0.265
**Stages**				
I II III IV	50(7.4%) 54(8.0%) 173(25.5%) 401(59.1%)	57(12.3%) 54(11.6%) 131(28.2%) 222(47.9%)	107 108 304 623	*<0*.001**
**Smoke status**				
No Yes	301(44.4%) 377(55.6%)	191(41.2%) 273(58.8%)	492 650	0.279
**Metastasis**				
**Brain**				
**No** Yes	595(87.8%) 83(12.2%)	422(91.0%) 42(9.0%)	1017 125	0.090
**Bone**				
No Yes	525(77.4%) 153(22.6%)	395(85.1%) 69(14.9%)	920 222	*<0*.05*
**Liver**				
No Yes	613(90.4%) 65(9.6%)	427(92.0%) 37(8.0%)	1040 102	0.348
**Adrenal gland**				
No Yes	639(94.2%) 39(5.8%)	442(95.3%) 22(4.7%)	1081 61	0.456
**Lymph node**				
No Yes	267(39.4%) 411(60.6%)	212(45.7%) 252(54.3%)	479 663	*<0*.05*
**Intrapulmonary**				
No Yes	594(87.6%) 84(12.4%)	419(90.3%) 45(9.7%)	1013 129	0.158
**Pleural**				
No Yes	579(85.4%) 99(14.6%)	404(87.1%) 60(12.9%)	983 159	0.423
**Mediastinal**				
No Yes	656(96.8%) 22(3.2%)	455(98.1%) 9(1.9%)	1111 31	0.183

*p values were calculated using the Chi-square test. (*P<0.05, **P<0.001)

LDH stratification analysis showed that an increased plasma LDH level was closely associated with gender (p < 0.05), with a decreased LDH level in female patients (33.5% of LDH-negative patients vs. 26.5% of LDH-positive patients) but an increased LDH level in male patients (66.5% of LDH-negative patients vs. 73.9% of LDH-positive patients). By histological analysis, it was shown that LDH was increased in SCLC patients (12.4% of LDH-negative patients vs. 19.2% of LDH-positive patients) but decreased in SCC patients (55.2% of LDH-negative patients vs. 49.8% of LDH-positive patients, p < 0.05). An increased plasma LDH level was also correlated to smoking history (54.6% of LDH-negative patients vs. 61% of LDH-positive patients, p < 0.05) (Table 3). According to the metastasis analysis, it was found that an enhanced LDH level was positively correlated with bone metastasis (16.5% of LDH-negative patients vs. 24.6% of LDH-positive patients, p < 0.05), adrenal gland metastasis (3.7% LDH-negative patients vs. 8.3% of LDH-positive patients, p < 0.05), lymph node metastasis (55.6% of LDH-negative patients vs. 62.4% of LDH-positive patients, p < 0.05), and mediastinal metastasis (1.9% of LDH-negative patients vs. 4.1% of LDH-positive patients, p < 0.05) (Table 3). Together, these results indicated that the plasma LDH level was closely related with the occurrence of lung cancer metastasis.

**Table 3:** Association of clinical characteristics and metastasis after stratification analysis by plasma LDH levels.

	Negative n = 732	positive n = 410	Total n = 1142	*p* value
**Basic Characteristic**				
**Age**				
<45 years 45–60 years >60 years	60(8.2%) 291(39.8%) 381(52.0%)	37(9.0%) 172(42.0%) 201(49.0%)	97 463 582	0.608
**Gender**				
Male Female	487(66.5%) 245(33.5%)	303(73.9%) 107(26.1%)	790 352	*<0*.05*
**Histological classification**				
Adenocarcinoma Squamous SCLC Others	202(27.6%) 404(55.2%) 91(12.4%) 35(4.8%)	105(25.6%) 204(49.8%) 79(19.2%) 22(5.4%)	307 608 170 57	*<0*.05*
**Stages**				
I II III IV	85(11.6%) 80(11.0%) 203(27.7%) 364(49.7%)	22(5.4%) 28(6.8%) 101(24.6%) 259(63.2%)	107 108 304 623	*<0*.001**
**Smoke status**				
No Yes	332(45.4%) 400(54.6%)	160(39.0%) 250(61.0%)	492 650	*<0*.05*
**Metastasis**				
**Brain**				
No Yes	662(90.4%) 70(9.6%)	355(86.6%) 55(13.4%)	1017 125	*<0*.05*
**Bone**				
No Yes	611(83.5%) 121(16.5%)	309(75.4%) 101(24.6%)	920 222	*<0*.001**
**Liver**				
No Yes	693(94.7%) 39(5.3%)	347(84.6%) 63(15.4%)	1040 102	*<0*.001**
**Adrenal gland**				
No Yes	705(96.3%) 27(3.7%)	376(91.7%) 34(8.3%)	1081 61	*<0*.05*
**Lymph node**				
No Yes	325(44.4%) 407(55.6%)	154(37.6%) 256(62.4%)	479 663	*<0*.05*
**Intrapulmonary**				
No Yes	659(90.0%) 73(10.0%)	354(86.3%) 56(13.7%)	1013 129	0.059
**Pleural**				
No Yes	639(87.3%) 93(12.7%)	344(84.0%) 66(16.0%)	983 159	0.112
**Mediastinal**				
No Yes	718(98.1%) 14(1.9%)	393(95.9%) 17(4.1%)	1111 31	*<0*.05*

*p values were calculated using the Chi-square test. (*P<0.05, **P<0.001)

### Correlation of CK or LDH with metastases of adenocarcinoma (ADC) and SCC

ADC and SCC were the two major histological classifications, accounting for 85% of the lung cancer. There were 608 ADC patients who were further separated into CK- or LDH-negative and -positive groups. CK stratification analysis, including 366 cases in the negative group and 242 cases in the positive group, showed that the plasma CK levels were increased in male patients (50.3% of CK-negative patients vs. 61.2% of CK-positive patients) and decreased in female patients (49.7% of CK-negative patients vs. 38.8% of CK-positive patients, p < 0.05) (Table 4). A decreased CK level was also closely related to increased bone metastasis (27.6% of CK-negative patients vs. 19.4% of CK-positive patients, p < 0.05). Although mediastinal metastasis analysis showed similar results as the bone metastasis analysis, the total number of cases was too small (only 15 cases) to draw a conclusion (Table 4). LDH stratification analysis showed that an increased LDH level was closely related to male patients, while a decreased LDH level was closely related to female patients, respectively. For stage analysis, it was found that the LDH level was low in stage I (12.9% of LDH-negative patients vs. 6.4% of LDH-positive patients) and stage II (10.1% of LDH-negative patients vs. 4.9% of LDH-positive patients) patients. However, the LDH level was increased dramatically in stage IV patients (57.2% of LDH-negative patients vs. 72.5% of LDH-positive patients, p < 0.05). In addition, an enhanced LDH level was associated with smoking history (37.6% of LDH-negative patients vs. 46.1% of LDH-positive patients, p < 0.05) (Table 5). For metastasis analysis, a high level of LDH was dramatically associated with an increased probability of bone (19.3% of LDH-negative patients vs. 34.3% of LDH-positive patients, p < 0.001), liver (4.8% of LDH-negative patients vs. 14.7% of LDH-positive patients, p < 0.001), and adrenal gland (2.2% of LDH-negative patients vs. 8.8% of LDH-positive patients, p < 0.001) metastases (Table 5), which clearly indicated the importance of LDH for predicting bone, liver, and adrenal gland metastases in ADC patients.

**Table 4:** Association of clinical characteristics and metastasis stratified by CK levels in adenocarcinoma patients.

	Negative n = 366	Positive n = 242	Total n = 608	*p* value
**Basic Characteristic**				
**Age**				
<45 years 45–60 years >60 years	43(11.7%) 139(38.0%) 184(50.3%)	24(9.9%) 89(36.8%) 129(53.3%)	67 228 313	0.684
**Gender**				
Male Female	184(50.3%) 182(49.7%)	148(61.2%) 94(38.8%)	332 276	*<0*.05*
**Stages**				
I II III IV	26(7.1%) 27(7.4%) 66(18.0%) 247(67.5%)	39(16.1%) 24(9.9%) 47(19.4%) 132(54.6%)	65 51 113 379	*<0*.05*
**Smoke status**				
No Yes	224(61.2%) 142(38.8%)	138(57.0%) 104(43.0%)	362 246	0.304
**Metastasis**				
**Brain**				
No Yes	306(83.6%) 60(16.4%)	216(89.3%) 26(10.7%)	294 86	0.050
**Bone**				
No Yes	265(72.4%) 101(27.6%)	195(80.6%) 47(19.4%)	460 148	*<0*.05*
**Liver**				
No Yes	334(91.3%) 32(8.7%)	225(93.0%) 17(7.0%)	559 49	0.446
**Adrenal gland**				
No Yes	349(95.4%) 17(4.6%)	232(95.9%) 10(4.1%)	581 27	0.764
**Lymph node**				
No Yes	148(40.4%) 218(59.6%)	119(49.2%) 123(50.8%)	267 341	*<0*.05*
**Intrapulmonary**				
No Yes	312(85.2%) 54(14.8%)	216(89.3%) 26(10.7%)	528 80	0.152
**Pleural**				
No Yes	294(80.3%) 72(19.7%)	199(82.2%) 43(17.8%)	493 115	0.557
**Mediastinal**				
No Yes	353(96.4%) 13(3.6%)	240(99.2%) 2(0.8%)	593 15	*<0*.05*

*p values were calculated using the Chi-square test. (*P<0.05, **P<0.001)

**Table 5:** Association of clinical characteristics and metastasis stratified by LDH levels in adenocarcinoma patients.

	Negative N = 404	Positive N = 204	Total N = 408	*p* value
**Basic Characteristic**				
**Age**				
<45 years 45–60 years >60 years	41(10.1%) 146(36.1%) 217(53.8%)	26(12.7%) 82(40.2%) 96(47.1%)	67 228 313	0.274
**Gender**				
Male Female	205(50.7%) 199(49.3%)	127(62.3%) 77(37.7%)	332 276	*<0*.05*
**Stages**				
I II III IV	52(12.9%) 41(10.1%) 80(19.8%) 231(57.2%)	13(6.4%) 10(4.9%) 33(16.2%) 148(72.5%)	65 51 113 379	*<0*.05*
**Smoke status**				
No Yes	252(62.4%) 152(37.6%)	110(53.9%) 94(46.1%)	362 246	*<0*.05*
**Metastasis**				
**Brain**				
**No** Yes	353(87.4%) 51(12.6%)	169(82.8%) 35(17.2%)	522 86	0.130
**Bone**				
No Yes	326(80.7%) 78(19.3%)	134(65.7%) 70(34.3%)	460 148	*<0*.001**
**Liver**				
No Yes	385(95.3%) 19(4.7%)	174(85.3%) 30(14.7%)	559 49	*<0*.001**
**Adrenal gland**				
No Yes	395(97.8%) 9(2.2%)	186(91.2%) 18(8.8%)	581 27	*<0*.001**
**Lymph node**				
No Yes	184(45.5%) 220(54.5%)	83(40.7%) 121(59.3%)	267 341	0.254
**Intrapulmonary**				
No Yes	356(88.1%) 48(11.9%)	172(84.3%) 32(15.7%)	528 80	0.190
**Pleural**				
No Yes	336(83.2%) 68(16.8%)	157(77.0%) 47(23.0%)	493 115	0.065
**Mediastinal**				
No Yes	397(98.3%) 7(1.7%)	196(96.1%) 8(3.9%)	593 15	0.100

*p values were calculated using the Chi-square test. (*P<0.05, **P<0.001)

A total of 307 patients were diagnosed as having SCC in this study. The results indicated that an increased plasma CK level was closely related to a decreased occurrence of bone metastasis (16.8% of CK-negative patients vs. 6.8% of CK-positive patients), while other metastases, including brain, liver, lymph node, et al., as well as clinical characteristics, such as age, gender, stage, and smoking history, showed no relationship with the CK level in SCC patients (S1 Table). It was also found that the LDH level was not related to clinical characteristics or metastasis occurrence in SCC patients (S2 Table).

### Both CK and LDH levels were related to survival in all lung cancer, ADC, and SCC patients

**Fig 1:**
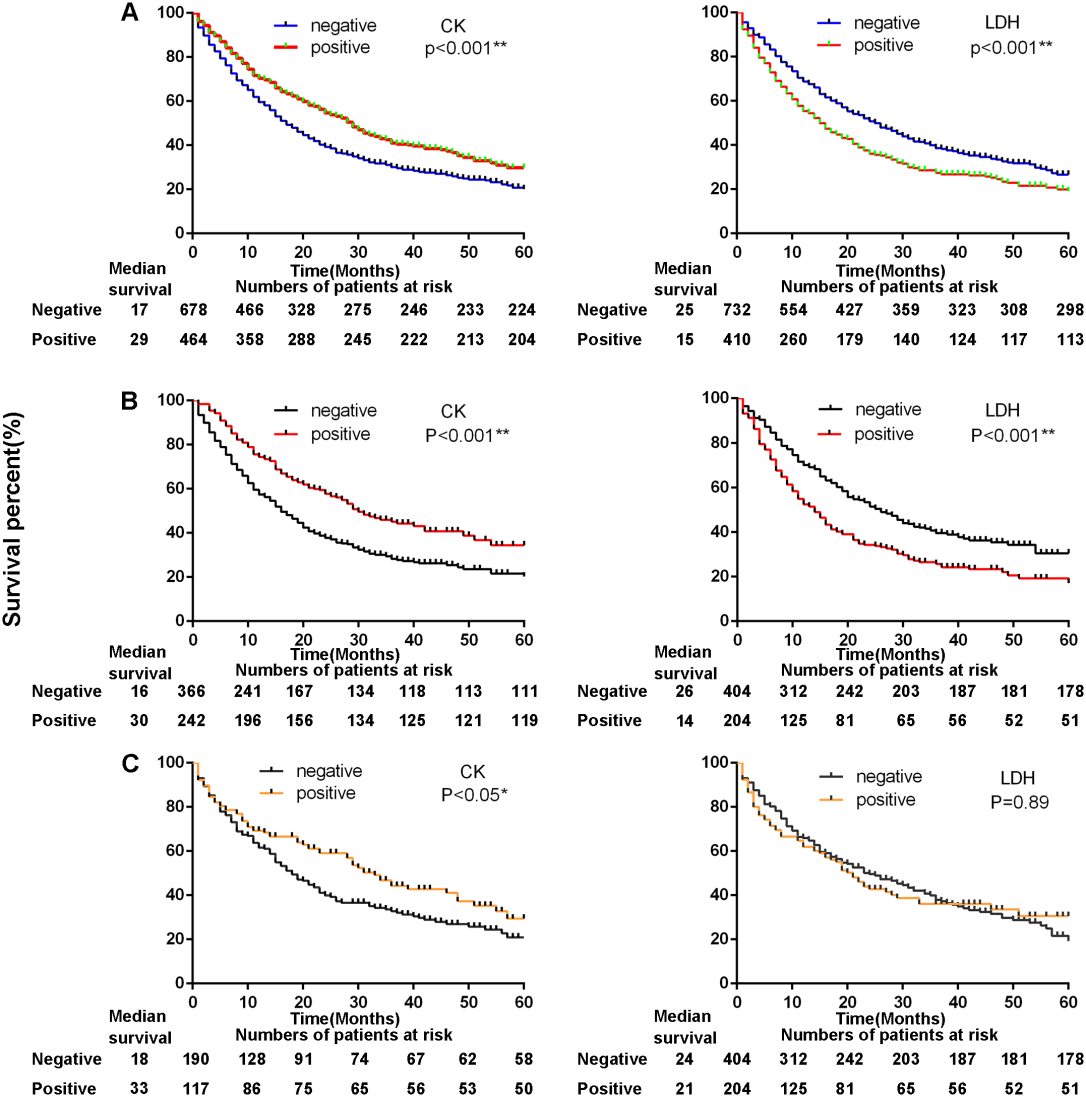
The survival status of non-small cell lung cancer patients correlated with CK and LDH. A, All lung cancer patients. Median observation period: CK (Negative 17 months;Positive 29 months); LDH (Negative 25 month;Positive 15 months). B, ADC patients. Median observation period: CK (Negative 16 months;Positive 30 months); LDH (Negative 26 month;Positive 14 months). C, SCC patients. Median observation period: CK (Negative 18 months;Positive 33 months); LDH (Negative 24 month;Positive 21 months). CK, creatine kinase; LDH, lactate dehydrogenase; ADC, adenocarcinoma; SCC, squamous cell carcinoma.

For all lung cancer patients (follow-up time range 1–60 months), the results indicated that a decreased plasma CK level was closely related to a poor survival status (p < 0.001) (Fig 1A). In addition, the patients with an increased LDH level showed poor outcomes (p < 0.001) (Fig 1A). It was also found that a decreased plasma CK level (p < 0.001) and an increased LDH level (p < 0.001) were strongly associated with a poor survival status in ADC patients (Fig 1B). However, the results confirmed that only the CK level was associated with the survival status in SCC patients (p < 0.05) (Fig 1C).

### Multivariate Cox regression analysis was conducted to identify poor prognostic factors

The multivariate Cox regression model was introduced to identify whether CK or LDH could be used as independent factors for predicting a poor prognosis in lung cancer patients. Our results found that the HR increased to 1.438 in patients aged older than 65 years old (95% CI: 1.091–1.895, p < 0.05) and to 1.195 in patients with a smoking history (95% CI: 1.028–1.389, p < 0.05) for all lung cancer patients. An advanced stage (III, HR: 2.171 and IV, HR: 2.842) and the occurrence of metastasis (HR: 1.326) also could serve as independent factors for predicting poor outcomes. For the two factors of CK and LDH used in this study, the HR decreased to 0.748 in the CK-positive group (95% CI: 0.643–0.871, p < 0.05), while it increased to 1.282 in the LDH-positive group (95% CI: 1.103–1.488, p < 0.05) (Table 6).

**Table 6:** Multivariate analysis for all lung cancer patients.

	Multivariate HR (95% CI)	*p* value
**Age**		
<45 45–65 >65	[1][Reference] 0.913(0.689–1.210) 1.438(1.091–1.895)	0.527 *<0*.05*
**Smokes**		
No Yes	[1][Reference] 1.195(1.028–1.389)	*<0*.05*
**Stages**		
I II III IV	[1][Reference] 1.377 (0.886–2.142) 2.171(1.481–3.182) 2.842 (1.956–4.130)	0.155 *<0*.001** <0.001**
**Metastasis**		
No Yes	[1][Reference] 1.326(1.094–1.607)	
**CK**		
Negative Positive	[1][Reference] 0.748(0.643–0.871)	*<0*.001**
**LDH**		
Negative Positive	[1][Reference] 1.282(1.103–1.488)	*<0*.05*

*p values were calculated using the Cox-proportional hazard model. (*P<0.05, **P<0.001)

Specific histological classification analysis was performed. The results were similar for ADC patients compared with all lung cancer patients. Moreover, an age ≥ 65 years old, a smoking history (HR: 1.228, 95%CI: 1.001–1.506), stage (II, HR: 2.149; III, HR: 4.267; IV, HR: 5.363), and metastasis (HR: 1.248, 95% CI: 0.941–1.656) could be used to predict a poor outcome in ADC patients. It was found that the HR decreased to 0.657 in the CK-positive group (95% CI: 0.529–0.815), while the HR increased to 1.442 in the LDH-positive group for the ADC patients (95% CI: 1.168–1.778) (Table 7). However, the results in this study revealed that CK and LDH could not be used as independent factors to predict a poor prognosis in SCC patients (Table 8). Therefore, our data only confirmed that CK and LDH were important for predicting poor outcomes in ADC patients.

**Table 7:** Multivariate analysis for ADC patients.

	Multivariate HR (95% CI)	*p* value
**Age**		
<45 45–65 >65	[1][Reference] 0.938(0.664–1.326) 1.373(0.983–1.919)	0.719 0.063
**Smokes**		
No Yes	[1][Reference] 1.228(1.001–1.506)	0.049
**Stages**		
I II III IV	[1][Reference] 2.149 (1.019–4.530) 4.267(2.219–8.202) 5.363 (2.845–10.110)	*<0*.05* <0.001** <0.001**
**Metastasis**		
No Yes	[1][Reference] 1.248(0.941–1.656)	0.125
**CK**		
Negative Positive	[1][Reference] 0.657(0.529–0.815)	*<0*.001**
**LDH**		
Negative Positive	[1][Reference] 1.442(1.168–1.778)	*<0*.05*

*p values were calculated using the Cox-proportional hazard model. (*P<0.05, **P<0.001)

**Table 8:** Multivariate analysis for SCC patients.

	Multivariate HR (95% CI)	*p* value
**Age**		
<45 45–65 >65	[1][Reference] 1.824(0.654–5.089) 3.832(1.387–10.586)	0.251 0.010
**Smokes**		
No Yes	[1][Reference] 0.819(0.573–1.172)	0.276
**Stages**		
I II III IV	[1][Reference] 0.857(0.442–1.660) 1.075(0.602–1.920) 1.106 (0.612–1.996)	0.647 0.808 0.739
**Metastasis**		
No Yes	[1][Reference] 1.765(1.241–2.512)	*<0*.05*
**CK**		
Negative Positive	[1][Reference] 0.742(0.547–1.005)	0.054
**LDH**		
Negative Positive	[1][Reference] 0.933(0.690–1.262)	0.652

*p values were calculated using the Cox-proportional hazard model. (*P<0.05, **P<0.001)

## Discussion

The present study had a large sample size to assess the association of LDH or CK levels with lung cancer prognosis. In this study, we found that CK and LDH were independently and closely related to metastasis occurrence and the survival status in lung cancer patients. In detail, CK was strongly associated with bone metastasis (p < 0.05) and lymph node (p < 0.05) metastasis, while LDH correlated with bone metastasis (p < 0.05), adrenal gland metastasis (p < 0.05), lymph node metastasis (p < 0.05), and mediastinal metastasis (p < 0.05). The lung cancer patients were then stratified by cancer type, ADC or SCC, according to the histological classification. It was found that CK was related to bone metastasis (p < 0.05) and mediastinal metastasis (p < 0.05) and that LDH was associated closely with bone metastasis (p < 0.001), liver metastasis (p < 0.001), and adrenal gland metastasis (p < 0.001) for ADC patients; however, only CK showed a relationship with bone metastasis (p < 0.05), while LDH did not show a relationship with metastasis or clinical characteristics for SCC patients. The Kaplan—Meier survival curves revealed that CK and LDH also showed a close relationship with the survival status for ADC patients but not for SCC patients.

Cox multivariate regression analysis indicated that CK and LDH could act as independent factors for predicting a poor prognosis in ADC patients. The HR decreased to 0.749 in the CK-positive group, while it increased to 1.281 in the LDH-positive group. According to specific subtype analysis, CK showed a low HR of 0.657 and LDH showed an HR of 1.385 in the corresponding positive groups for ADC patients. However, neither CK nor LDH were independent factors for a poor prognosis in SCC patients. Together, these results indicated the importance of CK and LDH in the diagnosis and prediction of a poor prognosis for ADC patients. In our study, CK and LDH were considered as auxiliary diagnostic factors. We believed that the diagnostic value of CK, LDH cannot be compared with the traditional biomarker CEA, Cyfra21-1, CYF, etc. So we just analyzed the value of CK and LDH in the diagnosis and prognosis of lung cancer.

LDH is an important enzyme that catalyzes the hydrogen transfer reaction between lactic acid and pyruvic acid [17], and it is widely distributed in various tissues of the body. The circulating level of LDH is elevated in malignant tumors [18]. The hypoxic microenvironment of a tumor can increase the conversion of pyruvic acid to lactic acid; therefore, unregulated LDH levels ensure proficient anaerobic glycolytic metabolism in the tumor environment, leading to reduced oxygen dependence [19]. An enhanced serum LDH level has been reported as a poor prognostic factor in patients with various malignant tumors, including breast cancer, T-cell lymphoma [20], pancreatic carcinoma [21], and renal cell carcinoma [22]. The LDH levels were significantly higher in metastatic stage IV lung cancer patients who were enrolled in a study of 329 lung cancer patients [23]. In addition, by analyzing 309 advanced-stage NSCLC patients treated with erlotinib, it was found that the change in LDH level during the first month was a surrogate marker for the efficacy of erlotinib in advanced NSCLC [24].

CK is an enzyme that is expressed by various cells and tissues and plays an important role in energy transduction in tissues, particularly in cardiac and skeletal muscle [25]. CK is one of the most important biomarkers that may reflect the status of innate immunity [26]. The study results indicated that the plasma CK levels were dramatically lower compared with positive group. The reason of plasma CK level decreased in lung cancer patients may be due to the CK played an important role in the growth of tumor cell and we hypothesized that growth tumor cells recruited circulating CK and finally resulted in the decline level of CK in circulation. Moreover, by analysis of 823 breast cancer patients, it was found that the serum CK levels were dramatically lower, compared with healthy controls, and were negatively correlated with the stage and tumor size of breast cancer [15].

Previously reported studies on LDH enrolled a relatively small patient sample size, comprising approximately 500 cases or fewer, and included no specific histological classification analysis such as ADC and SCC. To date, the correlation between CK and lung cancer has not been reported. In this retrospective analysis, 1142 patients were enrolled to analyze the correlation between the CK or LDH level with clinical characteristics, metastasis occurrence, as well as survival status. For the first time, our study analyzed the association between CK and lung cancer and confirmed the close relationship between CK and bone metastasis; systematically analyzed the role of LDH in metastasis occurrence and a poor outcome for lung cancer patients, particularly ADC and SCC patients; found that CK could act as an independent factor for predicting a poor prognosis in lung cancer patients, especially ADC patients; and confirmed that LDH is only closely related to a poor prognosis for ADC, not SCC.

The limitation of this study was that this was a one-center and retrospective study, and no consistent data were available to monitor the changes of CK and LDH levels with tumor progression. Furthermore, all analyses were conducted before surgery or other therapy; therefore, the relationship between the CK or LDH level and the therapeutic effects could not be evaluated.

In conclusion, Our results indicated that CK decreased while LDH increased in early stage of lung cancer, which suggested the diagnostic role of CK and LDH in lung cancer. At present, none of the biomarkers could be used alone for molecular diagnosis of tumors. Combination of multiple factors such as CEA, NSE, Cyfra21-1, as well as CK and LDH, together with imaging results, could provide more objective and accurate diagnosis. Plasma CK and LDH levels were identified as independent poor prognostic factors for ADC patients. The confirmation of these factors will help physicians to perform individualized treatment and interpret the contribution of CK and LDH to survival differences in clinical practice. Our results also suggest that further prospective studies should be performed with a large population size.

## Supporting information

S1 Table The association of clinical characteristics and metastasis occurrence between creatine kinase levels and squamous cell carcinoma patients. (DOCX)

S2 Table The association of clinical characteristics and metastasis occurrence between creatine kinase levels and squamous cell carcinoma patients. (DOCX)
