# Supplementary material for: Lactate dehydrogenase and creatine kinase as poor prognostic factors in lung cancer: A retrospective observational study
Source: PLoS One. 2017 Aug 2;12(8):e0182168. doi: 10.1371/journal.pone.0182168 (PMC5540491; doi:10.1371/journal.pone.0182168)
Supplement: S1 Table — (DOCX) [file pone.0182168.s001.docx]

**S1 Table. The association of clinical characteristics and metastasis occurrence between creatine kinase levels and squamous cell carcinoma patients**

|  | **Negative**  **N=190** | **positive**  **N=117** | **Total**  **N=307** | **P value** |
| --- | --- | --- | --- | --- |
| **Basic Characteristic** |  |  |  |  |
| **Age** |  |  |  |  |
| <45 years  45-60 years  >60 years | 8(4.2%)  71(37.4%)  111(58.4%) | 2(1.7%)  58(49.6%)  57(48.7%) | 10  129  168 | 0.074 |
| **Sex** |  |  |  |  |
| Male  Female | 172(90.5%)  18(9.5%) | 107(91.5%)  10(8.5%) | 279  28 | 0.784 |
| **Stages** |  |  |  |  |
| I  Ⅱ  Ⅲ  Ⅳ | 15(7.9%)  20(10.5%)  71(37.4%）  84(44.2%） | 12(10.3%)  21(17.9%)  45(38.5%）  39(33.3%） | 27  41  116  123 | 0.132 |
| **Smoke status** |  |  |  |  |
| No  Yes | 30(15,8%)  160(84.2%) | 26(22.2%)  91(77.8%) | 56  251 | 0.156 |
| **Metastasis** |  |  |  |  |
| **Brain** |  |  |  |  |
| No  Yes | 182(95.8%)  8(4.2%) | 112(95.7%)  5(4.3%) | 294  13 | 0.979 |
| **Bone** |  |  |  |  |
| No  Yes | 158(83.2%)  32(16.8%) | 109(93.2%)  8(6.8%) | 267  40 | ***<0.05**** |
| **Liver** |  |  |  |  |
| No  Yes | 173(91.1%)  17(8.9%) | 110(94.0%)  7(6.0%) | 283  24 | 0.347 |
| **Adrenal gland** |  |  |  |  |
| No  Yes | 180(94.7%)  10(5.3%) | 114(97.4%)  3(2.6%) | 294  13 | 0.254 |
| **Lymph node** |  |  |  |  |
| No  Yes | 78(41.1%)  112(58.9%) | 50(42.7%)  67(57.3%) | 128  179 | 0.772 |
| **Intrapulmonary** |  |  |  |  |
| No  Yes | 171(90.0%)  19(10.0%) | 106(90.6%)  11(9.4%) | 277  30 | 0.864 |
| **Pleural** |  |  |  |  |
| No  Yes | 173(91.1%)  17(8.9%) | 107(91.5%)  10(8.5%) | 280  27 | 0.904 |
| **Mediastinal** |  |  |  |  |
| No  Yes | 183(96.3%)  7(3.7%) | 115(98.3%)  2(1.7%) | 298  9 | 0.319 |

*p<0.05, **p<0.001
