# Supplementary material for: Lactate dehydrogenase and creatine kinase as poor prognostic factors in lung cancer: A retrospective observational study
Source: PLoS One. 2017 Aug 2;12(8):e0182168. doi: 10.1371/journal.pone.0182168 (PMC5540491; doi:10.1371/journal.pone.0182168)
Supplement: S2 Table — (DOCX) [file pone.0182168.s002.docx]

|  | **Negative**  **N=202** | **positive**  **N=105** | **Total**  **N=307** | **P value** |
| --- | --- | --- | --- | --- |
| **Basic Characteristic** |  |  |  |  |
| **Age** |  |  |  |  |
| <45 years  45-60 years  >60 years | 8(4.0%)  88(43.6%)  106(52.4%) | 2(2.0%)  41(39.0%)  62(59.0%) | 10  129  168 | 0.411 |
| **Sex** |  |  |  |  |
| Male  Female | 182(90.1%)  20(9.9%) | 97(92.4%)  8(7.6%) | 279  28 | 0.510 |
| **Stages** |  |  |  |  |
| Ⅰ  Ⅱ  Ⅲ  Ⅳ | 23(11.4%)  28(13.9%)  72(35.6%）  79(39.1%） | 4(3.8%)  13(12.4%)  44(41.9%）  44(41.9%） | 27  41  116  123 | 0.140 |
| **Smoke status** |  |  |  |  |
| No  Yes | 39(19.3%)  163(80.7%) | 17(16.2%)  88(83.8%) | 56  251 | 0.502 |
| **Metastasis** |  |  |  |  |
| **Brain** |  |  |  |  |
| **No**  Yes | 194(96.0%)  8(4.0%) | 100(95.2%)  5(4.8%) | 294  13 | 0.741 |
| **Bone** |  |  |  |  |
| No  Yes | 175(86.6%)  27(13.4%) | 92(87.6%)  13(12.4%) | 267  40 | 0.808 |
| **Liver** |  |  |  |  |
| No  Yes | 190(94.1%)  12(5.9%) | 93(88.6%)  12(11.4%) | 283  24 | 0.089 |
| **Adrenal gland** |  |  |  |  |
| No  Yes | 194(96.0%)  8(4.0%) | 100(95.2%)  5(4.8%) | 294  13 | 0.741 |
| **Lymph node** |  |  |  |  |
| No  Yes | 90(44.6%)  112(55.4%) | 38(36.2%)  67(63.8%) | 128  179 | 0.159 |
| **Intrapulmonary** |  |  |  |  |
| No  Yes | 184(91.1%)  18(8.9%) | 93(88.6%)  12(11.4%) | 277  30 | 0.481 |
| **Pleural** |  |  |  |  |
| No  Yes | 185(91.6%)  17(8.4%) | 95(90.5%)  10(9.5%) | 280  27 | 0.745 |
| **Mediastinal** |  |  |  |  |
| No  Yes | 198(98.0%)  4(2.0%) | 100(95.2%)  5(4.8%) | 298  9 | 0.170 |

**S2 Table. The association of clinical characteristics and metastasis occurrence between lactate dehydrogenase levels and squamous cell carcinoma patients**
